# A Tumor-Immune Interaction Model for Synergistic Combinations of Anti PD-L1 and Ionizing Irradiation Treatment

**DOI:** 10.3390/pharmaceutics12090830

**Published:** 2020-08-31

**Authors:** Jong Hyuk Byun, In-Soo Yoon, Yong Dam Jeong, Sungchan Kim, Il Hyo Jung

**Affiliations:** 1 Department of Mathematics, Pusan National University, Busan 46241, Korea; maticax@gmail.com (J.H.B.); wde0539@hanmail.net (Y.D.J.); scfrom88@daum.net (S.K.); 2 Institute of Mathematical Sciences, Pusan National University, Busan 46241, Korea; 3 College of Pharmacy, Pusan National University, Busan 46241, Korea; insoo.yoon@pusan.ac.kr; 4 Finance Fishery Manufacture Industrial Mathematics Center on Big Data, Pusan National University, Busan 46241, Korea

**Keywords:** immunotherapy, anti-PD-L1, ionizing irradiation, pharmacokinetics, tumor-immune interaction, global sensitivity, immuno-oncology, mathematical modeling

## Abstract

Combination therapy with immune checkpoint blockade and ionizing irradiation therapy (IR) generates a synergistic effect to inhibit tumor growth better than either therapy does alone. We modeled the tumor-immune interactions occurring during combined IT and IR based on the published data from Deng et al. The mathematical model considered programmed cell death protein 1 and programmed death ligand 1, to quantify data fitting and global sensitivity of critical parameters. Fitting of data from control, IR and IT samples was conducted to verify the synergistic effect of a combination therapy consisting of IR and IT. Our approach using the model showed that an increase in the expression level of PD-1 and PD-L1 was proportional to tumor growth before therapy, but not after initiating therapy. The high expression level of PD-L1 in T cells may inhibit IT efficacy. After combination therapy begins, the tumor size was also influenced by the ratio of PD-1 to PD-L1. These results highlight that the ratio of PD-1 to PD-L1 in T cells could be considered in combination therapy.

## 1. Introduction

Immunotherapy using immune checkpoint blockade (IT) is an anti-cancer therapy that recovers immunity by suppressing various tumor mechanisms that evade the immune response [1,2,3,4]. In particular, new ITs primarily aim to inhibit cytotoxic T-lymphocyte-associated protein 4 (CTLA-4), programmed cell death protein 1 (PD-1), and programmed death ligand 1 (PD-L1) to enable T lymphocytes to attack cancer cells [5,6,7]. Despite the impressive potential of these checkpoint blockade in the treatment of various cancers, this therapy strategy remains challenging because the response to a certain subgroup of IT is varied among the cancer patients [8,9]. In recent years, several studies have aimed to achieve synergistic effects through combination therapy to compensate for the shortcomings of monotherapy [10,11,12,13].

There are mathematical models of immune-tumor interactions to demonstrate the data of Deng et al. [14]. These studies [15,16] revealed the synergistic effect of anti-PD-L1 and IR combination therapy in mice. Chappell et al. [15] discussed that IT is directly involved in increasing T cell levels and indirectly stimulates tumor death via T cells. The underlying assumption considered that IR affects the death rate of both tumors and T cells. Based on this assumption, they formulated a mathematical model for the interaction between T cells and tumors. Model simulation was roughly conducted, focusing on the relationship between six compartments considering inhibition and activation. The main finding was the modeling and its equilibrium analysis; however, data fitting was not performed accurately. Additionally, the sensitivity analysis of the main parameters was relatively less investigated. Due to excessive suppression of IT and the bias of control growth, the synergistic effect of combined IR and IT was not accurately measured. Nevertheless, this model was able to explain how IT and IR affected tumors and T cells.

The other model was from Nikolopoulou et al. [16]. They considered PD-1 and PD-L1 expressed on the surface of tumors and T cells. The model was established through the assumption that tumors express PD-L1, and T cells express both PD-1 and PD-L1 on their surface. Negative feedback from the PD-1-PD-L1 complex occurs by the binding of PD-1 and PD-L1, resulting in inhibition of T cell growth. This model simplifies the binding process of PD-1 and PD-L1 by employing equilibrium assumptions. Thus, a three-compartment model was used. Anti-PD-L1 was composed of a system that reduced tumor size by suppressing the growth inhibition of T cells by inhibiting PD-L1. However, this model was less well verified by experimental data; anti-PD-L1 monotherapy was applied, but was less effective. Nevertheless, it is valuable to note the effect of immunotherapy on tumors or T cells through correlation with PD-1 and anti-PD-L1.

In this study, we proposed a mathematical model for tumor-immune interactions using anti-PD-L1 and IR in combination therapy. Here, we refer to anti-PD-L1 exclusively as IT. Deng et al. presented mice data for change in tumor size during combination therapy with anti-PD-L1 and IR. In the study, TUBO tumor cells from a spontaneous mammary tumor were injected subcutaneous injection (s.c.) into the flanks of mice. After allowing to grow for about two weeks, tumors were treated by IR with single dose and IT with administrated intraperitoneal injection to mice every three days for a total of three times. The experiment of control (without treatment), IR only, IT only and combination of IR and IT are conducted. They revealed that combination therapy had a synergistic effect that enhances host antitumor immunity and increases the efficacy of either treatment alone. Our mathematical model reproduced Deng et al.’s published data to describe tumor-T cell interaction through IT and IR, including PD-1 and PD-L1. Thus, this model enabled us to examine suppression of the tumor size by combination therapy and explore the effects of the ratio of PD-1 to PD-L1. Model simulation verified the data from combination therapy after estimating parameters using control, IT only, and IR only data. The synergistic effect was also confirmed through the model. Using global sensitivity, we measured the influence of essential parameters related to the change in PD-1 and PD-L1. Our approach showed that PD-1 or PD-L1 expression levels positively determined tumor size before therapy, but the sum of the expression level of PD-1 and PD-L1 was uncorrelated to tumor size after therapy has begun. Additionally, the high expression level of PD-L1 in T cells may inhibit IT efficacy. After combination therapy begins, the efficiency was influenced by the ratio of PD-1 to PD-L1. The model results revealed that the ratio of PD-1 to PD-L1 in T cells could be considered as one of the factors to determine the efficacy of combination therapy.

## 2. Materials and Methods

### 2.1. Assumptions

**Figure 1:**
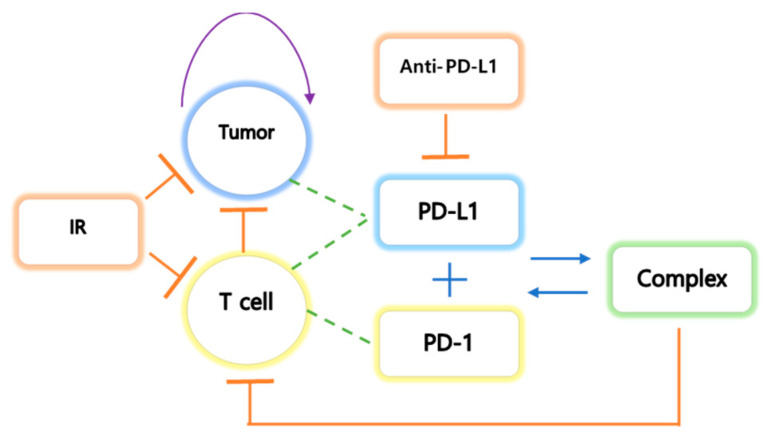
The tumor undergoes logistic growth, and T cells activate the cytokines interleukin 1 (IL1) and interleukin 12 (IL12). IL1 and IL12 are activated by stimulation of the tumor. The tumor expresses PD-L1 on the cell surface, and T cells express both PD-L1 and PD-1 on their surface. The binding process of PD-L1 and PD-1 results in negative feedback in T cells. IT inhibits the growth of both tumor and T cells and inhibits PD-L1 by preventing the formation of the PD-L1-PD-1 complex, which prevents the inhibition of T cell growth.

We have made the following assumptions. (i) Tumors express PD-L1 on the surface, and an increase in tumor size leads to an increase in the rate of the concentration of PD-L1. Additionally, an increase in CD8+ T cell number induces an increase in the rate of the concentration of PD-1 and PD-L1 because T cells express both PD-1 and PD-L1. (ii) The tumor grows logistically, and the natural death of the tumor is not considered. Tumor elimination depends on IT indirectly and IR directly. (iii) IT therapy is modeled using a pharmacokinetic two-compartment model that reflects administration by intraperitoneal injection (I.P.). The two-compartment model is also considered for IR therapy. IR therapy is administered immediately, but tumor suppression by IR is relatively delayed. The extra compartment reflects the delay in tumor inhibition by the therapy. The concentration in the tissue compartment is denoted by $A_{T}$. (iv) Volume, *V*, in mice is assumed to be 50 μL. (v) The initial density ($10^{9}$ cells/L) of T cells is $6\times 10^{-4}$, and the initial concentrations of PD-1 and PD-L1 are assumed to be $1\times 10^{-5}$. This assumption indicates that the initial density of T cells is small, and their concentrations begin to increase after the tumor is implanted. A schematic diagram of tumor-immune interactions is shown in Figure 1.

### 2.2. Data Derivation from the Study

**Figure 2:**
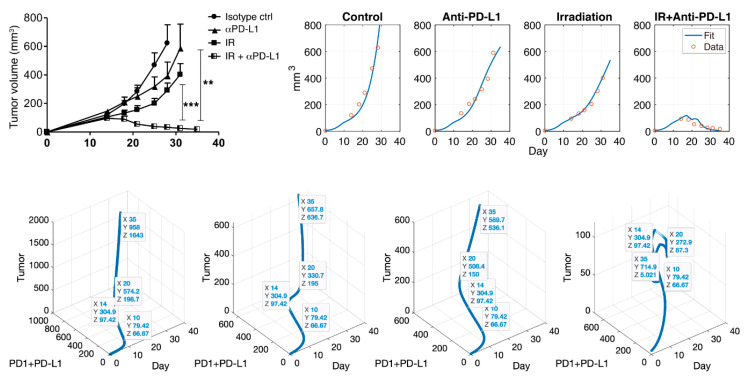
Note that α PD-L1 is IT (anti-PD-L1). Deng et al. showed the synergistic effect of combination therapy (top left). Simulation results (top right) after data fitting are presented through the mathematical model. This model captures the control, IT, and IR well, and this model also fits combination therapy. The blue curve indicates data fit using the Equations (1)–(6), and the red circle is experimental data. A scatter plot is shown for tumor vs. PD-1+PD-L1 vs. day (bottom). Each figure indicates control, IT, IR, and the combination of IR and IT in turn. Data tips are marked on day 10 (before therapy), 14 (beginning of IR and IT therapy), 20 (during IT therapy), and 35 (final timepoint). The sum of PD-1 and PD-L1 does not always positively correlate with tumor size after therapy. The coordinates X, Y, and Z, as shown in bottom figure, represent days, PD-1 + PD-L1 concentration and tumor size, respectively. **: *p* < 0.01; ***: *p* < 0.001.

Published data was obtained from the experiments of Deng et al. Their study was to verify the synergistic antitumor effects of IR and IT. A combination of IT and IR significantly enhanced the inhibition of TUBO growth in their study. The outline of the experiment process was as follows. BALB/c mice were inoculated by subcutaneous injection with $1\times 10^{6}$ TUBO cells (the size $5\;{\mathrm{mm}}^{3}$). After injected s.c. and waiting for 14 days (growing), tumors were locally treated with single 12 Gy of IR and 200 μg IT (antibody, clone 10F.9G2) by intraperitoneal injection every three days for a total of four doses. Tumor size was measured in mice with no therapy, IT only, IR only, and combination therapy with IT and IR. For reproducing the experimental data, the data values were extracted from the study. Adobe Illustrator is used to find the coordinates of published data. Data values, shown in Figure 2 (left top panel), are presented in Table 1. Deng et al.’s study was to verify the synergistic antitumor effects of IR and IT. A combination of IT and IR significantly enhanced the inhibition of TUBO growth in their study.

### 2.3. Mathematical Modeling

Let C, T, PL, PD, S, R, and A denote tumor (volume), the density of CD8+ T cells (T cells), PD-L1, PD-1, PD-1-PD-L1 complex (μg/μL), IR (Gy), and IT (μg/μL), respectively. The tumor grows logistically, and T cells are activated by the cytokines interleukin 12 (IL12) and are proliferated by interleukin 2 (IL2). Both IL12 and IL2 are activated by stimulation of the tumor. Among the several types of immune cells playing a role against cancer, we have focused only on T cells. IL-12 and IL-2 play a significant role in the activation and differentiation of the population of T cells [17]. The tumor expresses PD-L1 on the cell surface, and T cells express both PD-L1 and PD-1 on their surface. The binding process of PD-L1 and PD-1 induces negative feedback in T cells. IT inhibits the growth of both tumor and T cells and inhibits PD-L1 by preventing the formation of the PD-L1-PD-1 complex, which indirectly prevents the inhibition of T cell growth.

#### 2.3.1. Modeling Therapy with IR (R) and IT (A)

An exponential decay model was used to model for IR as follows:

(1) $$\frac{dR_{1}}{dt}=-k_{1}R_{1},\;\frac{dR}{dt}=k_{1}R_{1}-k_{R}R,\;R_{1}\left(14\right)=12\;\mathrm{and}\;R_{1}\left(t\right)=0\;\mathrm{if}\;t<14.$$

In the case of IT, a pharmacokinetic model was used with a two-compartment model as follows:

(2) $$\begin{array}{c} \frac{dA_{T}}{dt}=-\left(k_{T}+k_{el}\right)\;A_{T},\;\frac{dA}{dt}=k_{T}A_{T}-k_{A}A,\;A_{T}\left(t\right)=0,\\ \;\mathrm{if}\;t<14,\;A_{T}\left(14\right)=\frac{200}{V}. \end{array}$$

Furthermore, 200 μg IT is administered every three days for a total of four doses in a 50 μL volume after beginning IT on day 14.

#### 2.3.2. Compartmental Modeling of the Tumor(C), T Cell(T), PD-L1(PL), PD-1(PD), and Complex(S) 

$$\frac{dC}{dt}=k_{c}\left(1-\frac{C}{C_{max}}\right)\cdot C-\left(\frac{d_{TC}T}{T_{IC}+T}+\frac{d_{RC}R}{R_{IC}+R}\right)\cdot C.$$

The second term of the right-hand side (RHS) indicates tumor elimination by IT and IR therapy.

$$\frac{dT}{dt}=\left(k_{I12}+k_{I2}T\right)\frac{\kappa }{S_{IC}+k_{D}S}-\left(d_{T}+\frac{d_{RT}R}{R_{IC}+R}\right)T.$$

The first term of the RHS indicates that the PD-1-PD-L1 complex suppresses T cell growth. The second term of the RHS describes natural cell death and additional elimination by IR.

$$\frac{dPL}{dt}=\nu \cdot \;\left(\mu C+\eta T\right)-\frac{d_{APL}A}{A_{IC}+A}PL-k_{on}PL\cdot PD+k_{off}S-d_{PL}PL.$$

The first term of the RHS represents the increase in PD-L1 expression in tumors and T cells. The second term indicates inhibition of PD-L1 by IT. The third and fourth terms indicate the binding process of PD-1 and PD-L1. We assume that this reaction occurs according to the mass action law, indicating a one to one correspondence.

$$\frac{dPD}{dt}=\nu \left(1-\eta \right)T-k_{on}PL\cdot P+k_{off}S-d_{PD}PD.$$

In the first term of the RHS, η,  0≤η≤1 determines the ratio of PD-1 to PD-L1 induced by T cells. That is, PD-L1 is more expressed as η approaches one.

$$\frac{dS}{dt}=k_{on}PL\cdot P-k_{off}S.$$

#### 2.3.3. Model Reduction: Quasi Steady-State Approximation (QSSA)

We assume that PD-1-PD-L1 complex S is quickly saturated. Then equilibrium is reached within a short period of time, and so we may apply quasi-steady-state assumption (QSSA) and obtain $k_{on}PL\cdot P=k_{off}S$. Assume that $k_{D}=\frac{k_{on}}{k_{off}}.$ Then the system is simplified as follows.

(3) $$\frac{dC}{dt}=\underset{\mathrm{Tumor}\;\mathrm{growth}\;}{\underbrace{k_{c}\left(1-\frac{C}{C_{max}}\right)}}\cdot C-\left(\underset{\mathrm{Inhibition}\;\mathrm{by}\;T\;\mathrm{cells}}{\underbrace{\frac{d_{TC}T}{T_{IC}+T}}}+\underset{\mathrm{Inhibition}\;\mathrm{by}\;\mathrm{IR}}{\underbrace{\frac{d_{RC}R}{R_{IC}+R}}}\right)\cdot C,$$

(4) $$\frac{dT}{dt}=\left(\underset{\mathrm{Activation}\;\mathrm{by}\;\mathrm{IL}12}{\underbrace{k_{I12}}}+\underset{\mathrm{Growth}\;\mathrm{by}\;\mathrm{IL}2}{\underbrace{k_{I2}T}}\right)\underset{\mathrm{Inhibition}\;\mathrm{by}\;\mathrm{complex}}{\underbrace{\frac{\kappa }{S_{IC}+k_{D}PL\cdot PD}}}-\left(\underset{\mathrm{Death}}{\underbrace{d_{T}}}+\underset{\mathrm{Inhibition}\;\mathrm{by}\;\mathrm{IR}}{\underbrace{\frac{d_{RT}R}{R_{IC}+R}}}\right)T,$$

(5) $$\frac{dPL}{dt}=\underset{\mathrm{Growth}\;\mathrm{by}\;\mathrm{tumor}\;\mathrm{and}\;T\;\mathrm{cells}}{\underbrace{\nu \left(\mu C+\eta T\right)}}-\underset{\mathrm{Inhibition}\;\mathrm{by}\;\mathrm{IT}\;}{\underbrace{\frac{d_{APL}A}{A_{IC}+A}}}PL-\underset{\mathrm{Degradation}}{\underbrace{d_{PL}}}PL,$$

(6) $$\frac{dPD}{dt}=\underset{\mathrm{Growth}\;\mathrm{and}\;\mathrm{the}\;\mathrm{ratio}\;\mathrm{expression}\;\mathrm{level}\;\mathrm{of}\;\mathrm{the}\;T\;\mathrm{cells}}{\underbrace{\nu \cdot \left(1-\eta \right)}}T-\underset{\mathrm{Degradation}}{\underbrace{d_{PD}}}PD.$$

Descriptions or units of the estimated parameters and compartments are presented in Table 2.

## 3. Results

### 3.1. Simulation using the Cancer-Immune Model with IR and IT Therapy

Combination therapy with IT and IR significantly enhanced the inhibition of tumor growth in mice, as shown in Figure 2 (top left). The mathematical model consisting of (1)–(6) was used to fit the data, as shown in Figure 2 (top right). Data from the control (no therapy), IT only, and IR only samples were fitted. From three data fittings, we estimated parameter values, as shown in Table 2. MATLAB, MathWorks^®^, was used for model implementation with ODE45, and parameter estimation was conducted with the nonlinear least square method using MATLAB and Berkeley Madonna. Using the estimated parameters, the model captured the synergistic effect of the combination therapy consisting of IR and IT, as shown in Figure 2 (IR + IT). Additionally, the model failed to capture data around day 22. This is because repeated combination therapies cause a slightly overstated discrepancy in the model. However, the overall data fitting is reliable, and the model captures the efficacy of the combination therapy in mice.

In the control case, tumor size, PD-1 and PD-L1 expression were positively exponentially proportional. In this case, the regression (function) over time from day 0 to 35 was $f\left(t\right)=11.45e^{0.0052\cdot t}$ with $R^{2}=0.9975$. Cases were compared before and after therapy. Before therapy, the linear regression was f(t)=0.267t+22.27 with $R^{2}=0.8019$. Linearity was well followed after therapy begins (day 14) and the regression was f(t)=2.461t−859.8 with $R^{2}=0.8732$. In the case of IT, there was a positive correlation after initiating therapy. The linear regression was $f\left(t\right)=1.451t-315.7\;\mathrm{with}\;R^{2}=0.9891$. Although the growth rate was suppressed by IT, the relationship between IT and tumor growth still had a positive correlation. Likewise, in the case of IR, the growth rate was lower, and the linear regression was $f\left(t\right)=1.294t-343.4\;\mathrm{with}\;R^{2}=0.6277$, which shows positive correlation. However, there was a negative correlation after beginning combination therapy, in which the tumor size decreases due to the synergistic effect. The linear regression was $f\left(t\right)=-0.2029t+150.7\;\mathrm{with}\;R^{2}=0.9731$.

### 3.2. The Expression Levels of PD-1 and PD-L1

**Figure 3:**
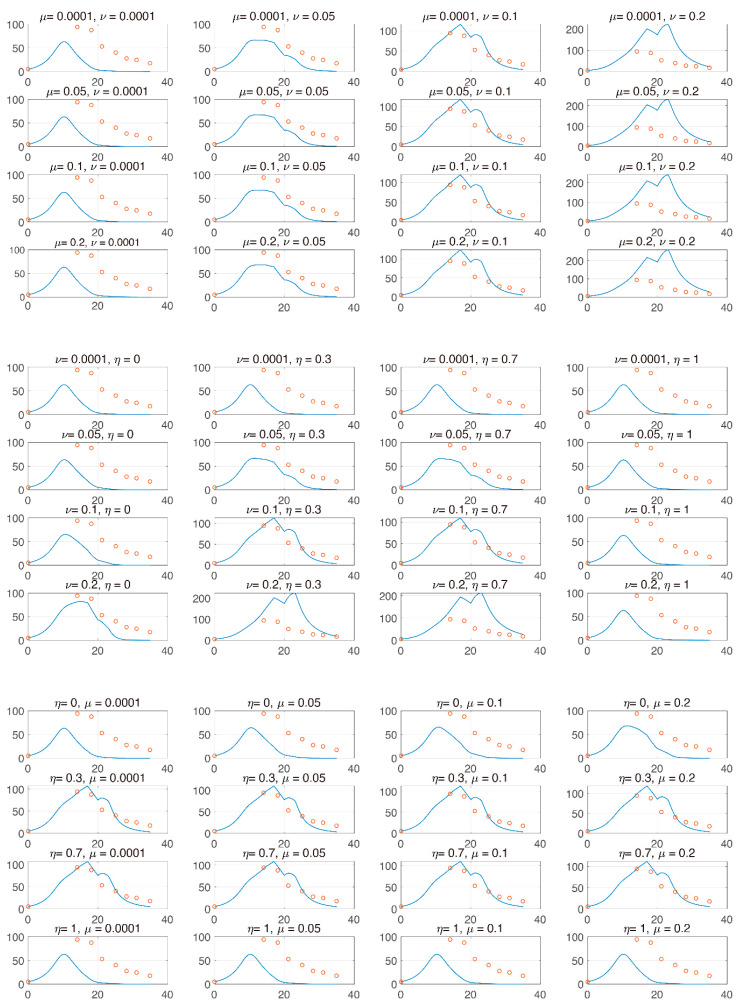
The x and y axes represent day and tumor size, respectively. Top panel: μ and ν are varied from [1 × 10^−4^, 0.2] and [1 × 10^−4^, 0.2], respectively. Center panel: μ and η are varied from [1 × 10^−4^, 0.2] and [0, 1], respectively. Bottom panel: η and μ are varied from [0, 1] and [1 × 10^−4^, 0.2], respectively. From these local changes, each parameter positively or negatively influences the change in tumor.

We utilized the model to explore which parameters cause tumor suppression among PD-1 and PD-L1. That is, we analyzed the changes in tumor size vs. parameters related to the changes in PD-1 and PD-L1. From (5)–(6), expression levels ν and μ determine the magnitude of the expression levels of PD-1 and PD-L1 on the surface of tumors and T cells. Given that T cells can express both PD-1 and PD-L1, the ratio η,  0≤η≤1, determines the ratio of the expression levels of PD-1 and PD-L1 in T cells. That is, if η is increasing, then so is the expression level of PD-L1 in T cells. Analyzing these parameters enables us to evaluate whether the concentration is more influential in removing the tumor among the expression levels of PD-1 and PD-L1. Specifically, we investigated how the IT efficacy is maximized depending on the expression level ratio of PD-1 to PD-L1. For this analysis, we attempted local changes in parameters, as shown in Figure 3, and two of the three parameters were compared. When modulating μ and ν (left panel), an increase in u and ν stimulates tumor growth. This is because an increase in PD-1 and PD-L1 under a fixed η causes T cell suppression, resulting in tumor growth. When modulating ν and η (middle), tumor growth depends on an increase in ν and decrease in η. This change indicates that IT could be more efficient when PD-1 is more highly expressed than PD-L1. When η and μ are varied (right), tumor growth depends on decreasing η and increasing μ. Thus, in the local sense, the decrease in η and the increase in ν and μ result in tumor growth. This indicates that IT efficacy is more potent when PD-1 level on T cells is smaller.

**Figure 4:**
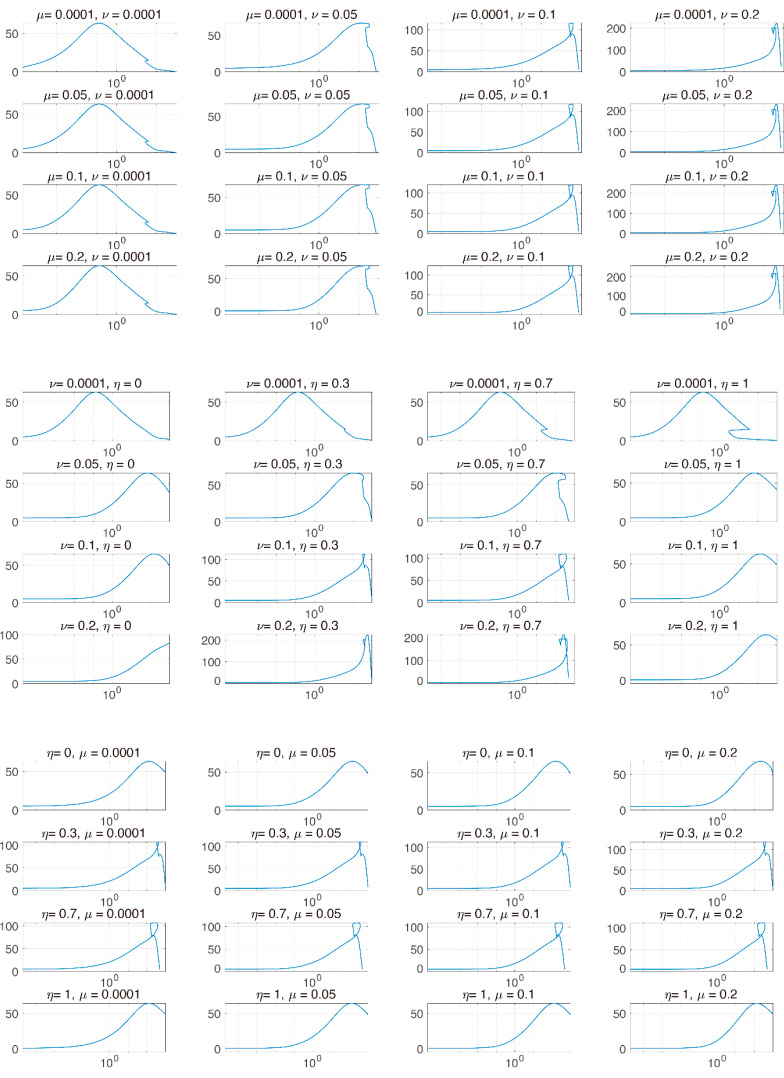
The x and y axes represent the sum of PD-1 and PD-L1 and tumor size, respectively. The x axis is plotted on a logarithmic scale. Each graph indicates the two parameters that are varied. μ, ν, and η are varied from [1 × 10^−4^, 0.2], [1 × 10^−4^, 0.2], and [0, 1], respectively. After beginning combination therapy, the sum of PD-1 and PD-L1 is not positively proportional to tumor size.

In Figure 4, we investigated tumor size vs. the sum of PD-1 and PD-L1 expression. We noticed that the total expression level of PD-1 and PD-L1 does not positively correlate tumor size when the tumor is inhibited by combination therapy, unlike IT and IR only. We inferred that this was because the balance of PD-1 and PD-L1 was broken, leading to a reduced concentration of the complex formed by PD-1 binding to PD-L1 and ultimately blocking T cell inhibition. However, these local changes in parameters could have biased influences given that these processes naturally assume that the other parameters remain constant. In particular, the change in expression levels of PD-1 and PD-L1 are simultaneously affected by various parameters.

Performing a two-parameter comparison could induce prediction bias. Thus, global sensitivity analysis is required to analyze how all parameters associated with the changes in PD-1 and PD-L1 are simultaneously changed.

From Figure 3 and Figure 4, the model verified that the magnitude of tumor reduction was changed according to the change in the parameter, and the change in tumor size due to the change in the sum of PD-1 and PD-L1 expression was compared. The model showed that the concentration of the sum of PD-1 and PD-L1 was not proportional to tumor size after therapy begins, indicating that if tumor size decreases, the correlation between them is deregulated. We believe that the synergistic effect of combination therapy accelerated the dysregulation of this correlation. However, further studies on the correlation between tumor size and the sum of PD-L1 and PD-1 expression should be conducted with more data.

The challenge is to determine how uncertainty in the output of a model can be apportioned to different sources of uncertainty in the model input (factors) [18]. Local methods analyze sensitivity around some (often optimal) points in the factor space, whereas global methods attempt to analyze variability across the full factor space. Changing one factor at a time means that whatever effect is observed on the output is due solely to that factor. Conversely, a non-zero effect implies influence; i.e., it never detects uninfluential factors as relevant. Local sensitivity has the above disadvantage; therefore, we performed global sensitivity analysis and analyzed tumor changes under the influence of other variables. The elimination rate $k_{el}$ is contained to evaluate the influence. Providing $k_{el}$ enables comparison to the influence of a dummy-parameter not directly related to PD-1 and PD-L1.

### 3.3. Global Sensitivity Analysis of the Parameter Space: Latin Hypercube Sampling Partial Rank Correlation Coefficient

We evaluated the influence of the parameters on the tumor size using global sensitivity analysis. Influences of the parameters were measured at days 10, 14, 20, and 35, using the partial rank correlation coefficient (PRCC) [18,19] based on Latin hypercube sampling (LHS) [20,21]. LHS is a sampling method that provides an unbiased estimation of the output of a model while requiring fewer samples to achieve the same accuracy of simple random sampling. For parameters $x_{j},\;j=1,2,\cdots ,k$ and tumor y, a correlation coefficient (CC) between $x_{j}$ and y is calculated as

$$r_{x_{j}y}=\frac{Cov\left(x_{j},y\right)}{\;\sqrt{Var\left(x_{j}\right)Var\left(y\right)}},$$

where Cov(x,y) is the covariance between $x_{j}$ and y, and $var\left(x_{j}\right)$ is the variance of $x_{j}$. CC varies between −1 and 1. Pearson correlation coefficient (PCC) characterizes the linear relationship considering residuals $x_{j}-\hat{x_{j}}$ and $y-\hat{y}$, where $\hat{x_{j}}=c_{0}+\sum_{i=1,i\neq j}^{k}c_{i}x_{i}$ and $\hat{y}=b_{0}+\sum_{i=1,i\neq j}^{k}b_{i}x_{i}.$ PRCC performs PCC, with $x_{j}$ and y being first-rank transformed parameters and tumor size is determined by LHS, respectively.

**Figure 5:**
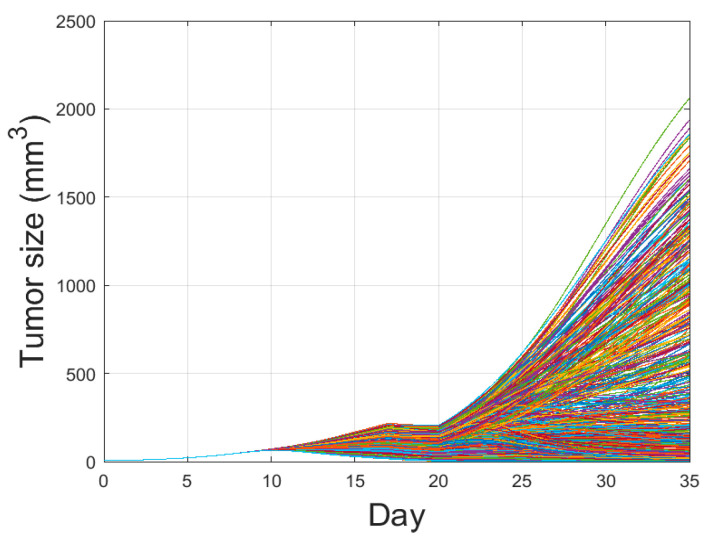
Tumor growth curve with combination therapy of IR and IT is plotted with the parameter variations using Latin Hypercube Sampling. One thousand cases with four parameters randomly extracted from (7) were conducted. By varying these parameters, some tumors are eliminated, some reach maximal tumor sizes, and the others are in between the two extremes. The amount of combination therapy and other parameter values were unchanged except for η, ν, μ, and $k_{el}.$

**Figure 6:**
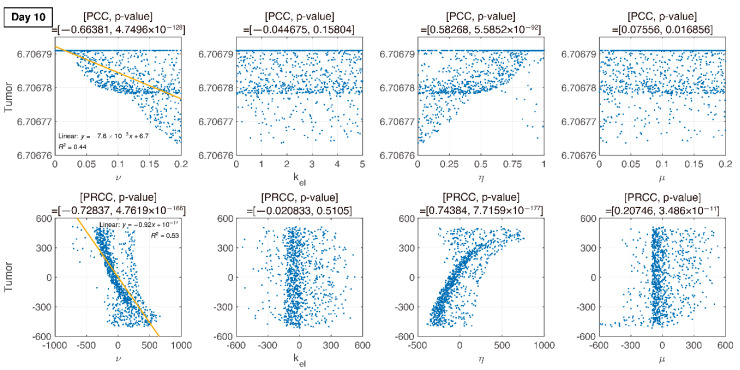
PCC and PRCC scatter plots of tumor size versus parameters $k_{el},\;\mu ,\;\nu ,\;\mathrm{and}\;\eta$ on day 10. All four parameters are varied simultaneously. The sample size is 1000. The x-axis represents the parameter values in PCC or the residuals of the linear regression between the rank-transformed values of the parameters. The y-axis represents the tumor size in PCC or the residuals of the linear regression between the rank-transformed values of tumor versus the rank-transformed values of the parameters. The title of each plot represents the PRCC value with the corresponding *p*-value. The linear relationship between parameters and tumor variation becomes more apparent with PRCC compared to PCC.

**Figure 7:**
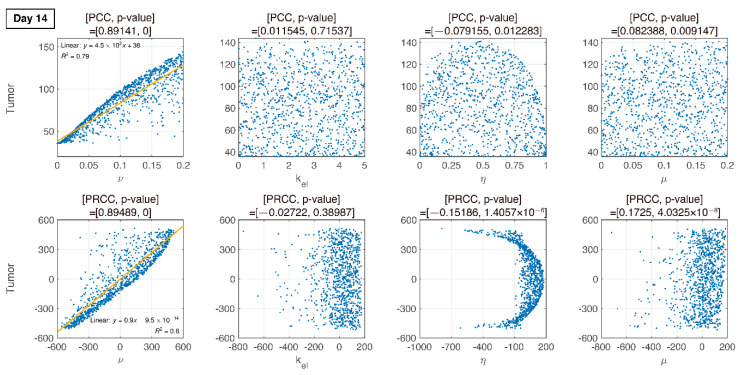
Dotted points indicate tumor size on day 14. Combination therapy begins on day 14. An increase in ν is proportional to tumor growth with PRCC and *p*-value. Other parameters are less influential.

**Figure 8:**
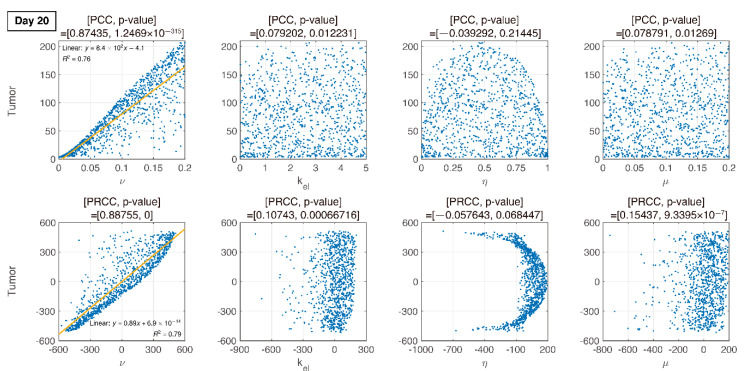
On day 20 (during IT therapy and after IR therapy), PRCC and *p*-values of four parameters are determined. An increase in ν is proportional to tumor growth, but other parameters have little influence.

**Figure 9:**
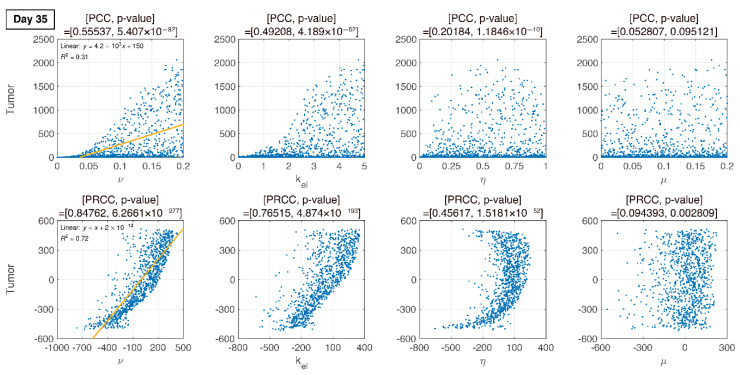
In the final phase, the elimination rate of IT, $k_{el},$ is proportional to tumor growth. ν remains proportional to tumor growth.

PRCC is a robust sensitivity measure for monotonic relationships between input and output. PRCC performs a partial correlation analysis on rank-transformed data in two steps: (i) input and output data are first rank-transformed, and then (ii) the linear regression model is applied. LHS is used for parameter sampling, in which each parameter is assumed to be uniformly distributed. We explored four parameters related to the changes in PD-1 and PD-L1 stimulated by the tumor and T cells. Herein, tumor size over the parameters is plotted using LHS, as shown in Figure 5. By varying the parameters associated with the changes in PD-1 and PD-L1, tumor size increases or decreases. The number of sampling parameters was 1000, and the ranges of the parameters were as follows:

(7) $$\begin{array}{c} \nu \sim U\left(1\;\times 10^{-5}\;,\;0.2\right),k_{el}\sim U\left(1\times 10^{-5},\;5\right),\eta \sim U\left(1\times 10^{-8},\;1\right),\\ \mu \sim \left(1\times 10^{-5},\;0.2\right), \end{array}$$

where U is uniform distribution. The resultant PRCCs with *p*-values are measured in Figure 6, Figure 7, Figure 8 and Figure 9 using LHS. This was done at the following timepoints: day 10 (before therapy), 14 (beginning of therapy), 20 (during therapy), and 35 (after therapy). Additionally, PCC was calculated for comparison. PCC is intuitive because there is no change in parameters and tumor size, but the linear relationship of PRCC between variations in parameters and tumor size becomes more apparent compared to PCC.

### 3.4. Analysis of Changes in Parameters

We compare PCC and PRCC with *p*-values, as shown in Figure 6, Figure 7, Figure 8 and Figure 9. PCC directly compares tumor size vs. parameters. While PCC is intuitive, it has a disadvantage in that the relationship between parameters and tumor size is not clearly identified. Therefore, PRCC is performed to examine the influence of parameters more clearly by transforming the value. Ultimately, the global sensitivity analysis investigates the changes in essential outputs caused by simultaneous changes in parameters. We explored the parameter influence at each critical timepoint. In particular, the change in parameters can be compared before and after therapy. The influence of $k_{el}\;\mathrm{and}\;\nu$ did not significantly affect both tumor growth and inhibition at day 10, before therapy. In the case of ν, negative linearity is observed in PRCC as compared to that in PCC. In the case of η, the linearity is not observed in PCC, but PRCC captures positive linearity. When combination therapy begins on day 14 (Figure 7), $k_{el},\;\eta$ and μ are not correlated to the change in tumor size. Tumor size has a positive linear relationship to ν, unlike before therapy. In Figure 8, which shows day 20 of IT therapy, tumor size and ν have a positive relationship, which becomes more apparent compared to PCC. After therapy (day 35), ν and $k_{el}$ are positively correlated with tumor size, as shown in Figure 9. This correlation indicates that the elimination rate of IT is influential to the change in tumor size when the amount of IT is small. From global sensitivity analysis, we may conclude that parameter influence over tumor size is different before and after therapy. These levels accelerated tumor inhibition after therapy began. Particularly, η determining the ratio of PD-1 to PD-L1 does not follow the linear relationship to tumor size.

Changes in tumor size with respect to ν before and after therapy were investigated. There was a negative correlation in the absence of therapy (before day 14), as shown in Figure 6, Figure 7, Figure 8 and Figure 9. However, a positive correlation was observed after IT, IR, and combination therapy began. This seemingly contradictory result demonstrated that, prior to initiating therapy, PCC showed a small change in tumor size (less than 3E-5), so the negative correlation may not have a significant effect on the change in tumor size. PRCC showed this difference to be more exaggerated, indicating that it was essential to examine both PCC and PRCC. Changes in ν after 14 days of therapy were positively correlated with tumor size. This relationship was due to the negative feedback by which the increase in ν caused by T cells leads to an increase in PD-L1, and a decrease in T cells by increasing the amount of PD-1 and PD-L1 binding. This mechanism increased tumor size and suggested that high PD-L1 expression levels in T cells could inhibit the efficiency of IT. Likewise, an increase in expression level ν⋅μ expressed on tumor cells caused an increase in PD-L1 regardless of therapy. Additionally, an increase in ν⋅μ resulted in an increase in tumor size for similar reasons.

## 4. Discussion and Conclusions

Localized IR has been mediated tumor reduction in a T cell-dependent fashion. Therapeutic blockade of the T cell negative regulator PD-L1 could enhance T cell effector function when PD-L1 was expressed in tumors, as it happens in the tumor microenvironment after IR [22,23]. PD-L1 expression in the tumor microenvironment provided an opportunity for therapeutic intervention using immunotherapies such as anti PD-L1, anti PD-1 and CTLA-4 [24,25].

The purpose of this work was to obtain insight into mechanisms of the tumor-immune interactions while combination therapy of IR and IT was conducted. Notably, we considered PD-1 and PD-L1 in the model to discuss how these targets change. Mathematical modeling is one of the most important tools in analyzing the characteristics of the synergistic combinations and providing some useful insights about the dynamic of the interactions. A mathematical model for the tumor-immune interactions was developed. QSSA was used to simplify the model. Additionally, two-compartment models for treatment were considered to reflect the delay of tumor repression. The simulation was implemented for control, IT only, and IR only data. From the estimated parameters, combination therapy with IR and IT was fit. Simulations of our model are shown to be in reliable agreement with mouse experiments for all cases [14].

The model addressed how tumors and T cells were inhibited or stimulated by changes in PD-1 and PD-L1 expression. To this end, we utilized global sensitivity analysis to examine the influence of four parameters and confirmed the corresponding changes in tumor size. It was possible to employ a model to investigate how T cells and tumor growth were stimulated by PD-L1 and PD-1. The model predicted that T cells would have a significant influence on tumor growth and inhibition based on the ratio of PD-1 to PD-L1 when IT is applied. This result was explored in the global sensitivity analysis of the parameter changes, rather than with local changes. We compared PCC and PRCC from LHS among global sensitivities. PCC was intuitive, but the relationship between parameters and tumor size variation was more apparent when assessed with PRCC than PCC.

Our approach complemented Deng et al.’s experiments that combination therapy of IR and IT was likely an important part of the complex regulation in the IR tumor microenvironment that suppresses antitumor immunity. Two models can be contrasted. The model of Nikolopoulou et al. was a system of three ODEs with 19 parameters. They theoretically investigated the change in tumors against anti-PD-1. Their major contribution was to predict the general behavior of the tumor-immune response to therapy. Sensitivity analysis using PRCC via LHS and bifurcation analysis for tumor and T cells were also conducted. The model of Chappell et al. was a system of 4 ODEs with 16 parameters. Their study concerned tumor-T cell interactions with combination therapy of IR and IT. Regardless of the consideration of PD-1 and PD-L1, one of their contributions was reproducing the synergistic effects of IR and IT. Theoretical analysis such as model equilibria and stability was also conducted. Our approach reflected those models and additionally focus on the change in the expression levels of PD-1 and PD-L1. However, the proposed model has some limitations. We assumed that PD-L1 is permanently expressed on tumor cells and the expression increases with a tumor progression. However, PD-L1 is heterogeneously expressed on cancer cells and can be decreased over a period. Furthermore, we did not consider other immune checkpoint molecules upregulated on cancer cells that also have a significant impact on the cancer cell immune escape.

In Figure 6, Figure 7, Figure 8 and Figure 9, the ratio rate η determined the ratio of PD-1 to PD-L1 in T cells. Before therapy, η and tumor size were positively correlated. That is, in our model, after cancer cells were implanted and before therapy, the binding of PD-1 to PD-L1 was small, and as a result, T cell suppression was small and the increase in tumor size was minimal, as shown in the experimental data (Figure 6). The linearity was deregulated after beginning combination therapy (Figure 7, Figure 8 and Figure 9). IT depleted PD-L1 to reduce the amount of binding to PD-1, thereby reducing T cell suppression and consequently suppressing tumors. IR additionally eliminated tumors and reduced the amount of PD-L1. Therefore, tumor size was determined based on T cell decrease/increase, where a decrease was caused by IR directly or an increase was caused by inhibiting the binding of PD-1 to PD-L1 indirectly.

Interestingly, as η decreased, the tumor size variation became more severe. The smaller the differentiation by T cells to PD-L1 was, the smaller the amount of PD-L1 was, and thus tumor growth was suppressed by IT. Meanwhile, when η was small with sufficient PD-L1, T cells increased the expression level of PD-1, thereby increasing the concentration of the PD-1-PD-L1 complex. As a result, the proliferation of the T cells was suppressed, and the tumor size increased. Therefore, two opposite cases can occur. Combination therapy overcomes the challenge by inhibiting the tumor both directly and indirectly. Using this model, we examined ways to regulate PD-L1 and PD-1 to improve the efficacy of combination therapy. After reducing PD-L1 and PD-1 expression by inhibiting tumor and T cells through IR, further depletion of PD-L1 through IT resulted in a decrease in the amount of PD-1-PD-L1 complex, thereby preventing the inhibition of T cell growth. This result indicated that if PD-L1 is lowly expressed in T cells, the efficacy of IT improves. That is, the level of PD-1-PD-L1 complex can be lower by reducing η.

It was noted that when η was small (i.e., when PD-1 was highly expressed in T cells or PD-L1 expression was low in T cells) and PD-L1 expression on the tumor was high, the tumor could be less inhibited as the PD-1-PD-L1 complex concentration was increased, indicating that T cell growth may be further suppressed. Additionally, combination therapy should be considered in two cases: when PD-L1 expression is high and when PD-1 expression is low or high in T cells. The expression level of PD-L1 was determined based on combination therapy. That is, when the expression level of PD-1 in T cells is low, successful therapy can be achieved by suppressing PD-L1 alone. Still, in the case of high expression of PD-L1 in T cells, such a strategy may fail due to excessive T cell reduction, although the tumor size was also reduced.

We investigated the tumor-immune interaction through a mathematical model. Through combination therapy, we verified the synergistic effect of combined IR and IT after parameter estimation through data fitting. Our model analyzed the mechanism of tumor inhibition through the relationship between PD-1 and PD-L1. We also examined the influence of each parameter through global sensitivity analysis of crucial parameters associated with the change in PD-1 and PD-L1. However, this analysis assumed a uniform distribution considering a slight variation in the parameters. Importantly, this assumption could not suggest a feasible condition for the parameters. In the next study, we will provide the distribution of the parameter space using Bayesian inference. This study could be valuable in that the Bayesian inference may be used for consideration of multiple doses and combination therapy remains challenging in the tumor-immune system.

## Figures and Tables

**Table 1:** Experimental data. Here, the control means without any therapy. IR therapy occurs on day 14 with 12 Gy, and IT therapy begins on day 14 and continues every three days for a total of four doses with 200 μg. Both indicate a combination therapy of IR and IT and each column represents time and tumor size, respectively.

Control		IR		IT (Anti-PD-L1)		Both	
0	5	0	5	0	5	0	5
13.8587	120.487	14.0761	95.8553	13.9674	135.2759	14.0761	94.2126
18.0435	202.7944	18.0435	133.8006	18.2065	206.0865	18.0977	87.8069
21.0326	289.9808	21.087	158.5662	21.3043	242.3534	21.1413	53.0777
25.0543	472.4868	24.8913	204.7183	24.9457	316.4249	25.1087	40.0989
28.0977	628.6693	28.1522	303.4149	31.1413	591.012	28.2065	27.7271
-	-	30.9783	402.0936	-	-	31.087	24.56
-	-	-	-	-	-	34.9457	17.505

**Table 2:** Estimated parameters and initial values used in the model are presented together with their descriptions and units.

Initial/Parameters	Description and Units	Estimated Values
C(0)	Tumor initial volume (${\mathrm{mm}}^{3}$)	5
T(0)	Initial lymphocytic density of CD8+ T cells ($10^{9}\;\mathrm{cells}/L$)	6 × 10^−4^ (Assumed)
PL(0)	Initial concentration of PD-L1 (μg/μL)	1 × 10^−5^ (Assumed)
PD(0)	Initial concentration of PD-1 (μg/μL)	1 × 10^−5^ (Assumed)
$A_{t}\left(14\right)$	Initial concentration of Anti-PD-L1 (μg/μL) in tissue	4
A(0)	Concentration of anti-PD-L1 (μg/μL)	0
$R_{1}\left(14\right)$	Irradiation (Gy)	12
$k_{c}$	Tumor growth rate ($1/{\mathrm{mm}}^{3}/\mathrm{day})$	0.29428
$C_{max}$	Maximum tumor size (${\mathrm{mm}}^{3}$)	3 × 10^3^
$d_{TC}$	Maximum tumor death rate by T cells (1/day)	0.53643
$T_{IC}$	Half maximum density of T cells $\left(10^{8}\;\mathrm{cells}/L\right)$	1 × 10^3^
$d_{RC}$	Maximum tumor death rate by irradiation (1/day)	0.5
$R_{IC}$	Half maximum irradiation (Gy)	8
V	Volume in mice (μL)	50
$k_{I12}$	T cell activation rate by cytokine IL12 $\left(10^{8}\;\mathrm{cells}/L/\mathrm{day}\right)$	10
$k_{I2}$	T cell proliferation rate by cytokine IL12 (1/day)	1 × 10^2^
$k_{D}$	Equilibrium constant (1/μg/μL)	1
κ	Inhibition constant of PD-L1 and PD-1 (μg/μL)	10
η	Expression level ratio of PD-L1 to PD-1 in T cells (unitless)	0.5
$S_{IC}$	Half maximum inhibition of PD-L1 and PD-1 (μg/μL)	1 × 10^3^
$d_{T}$	T cell death rate (1/day)	0.1
$d_{RT}$	Maximum T cell death rate by irradiation (1/day)	1
ν	Expression level of PD-L1 on activated T cells ($10^{3}\;\mu g/\mathrm{cell}/\mathrm{day})$	0.1
$d_{PL}$	Degradation rate of PD-L1 (1/day)	1 × 10^−2^
$d_{PD}$	Degradation rate of PD-1 (1/day)	1 × 10^−2^
μ	Expression level of PD-L1 by tumor vs. T cells $\left(\mathrm{cell}/\mu L/{\mathrm{mm}}^{3}\right)$	0.1
$d_{APL}$	Maximum PD-L1 inhibition rate by anti-PD-L1 (1/day)	20
$A_{IC}$	Half-maximum inhibition (μg/μL)	1
$k_{T}$	Intercompartment distribution rate (1/day)	1.5 × 10^−3^
$k_{el}$	Elimination rate of anti-PD-L1 in tissue (1/day)	0.1
$k_{A}$	Elimination rate of anti-PD-L1 (1/day)	0.05
$k_{1}$	Delay rate with the mean duration $1/k_{1}\;$(1/day)	0.15
$k_{R}$	Elimination rate of ionizing irradiation (1/day)	0.09
